# Nucleotide substitution rates of diatom plastid encoded protein genes are positively correlated with genome architecture

**DOI:** 10.1038/s41598-020-71473-1

**Published:** 2020-09-01

**Authors:** Yan Ren, Mengjie Yu, Wai Yee Low, Tracey A. Ruhlman, Nahid H. Hajrah, Abdelfatteh El Omri, Mohammad K. Alghamdi, Mumdooh J. Sabir, Alawiah M. Alhebshi, Majid R. Kamli, Jamal S. M. Sabir, Edward C. Theriot, Robert K. Jansen, Irfan A. Rather

**Affiliations:** 1 The Davies Research Centre, School of Animal and Veterinary Sciences, University of Adelaide, Roseworthy, SA 5371 Australia; 2 Department of Integrative Biology, University of Texas at Austin, Austin, TX USA; 3 Center of Excellence for Bionanoscience Research, King Abdul Aziz University, Jeddah, Saudi Arabia; 4 Department of Biological Sciences, Faculty of Science, King Abdulaziz University, Jeddah, Saudi Arabia; 5 Department of Information Technology, Faculty of Computer Science and Information Technology, King Abdul Aziz University, Jeddah, Saudi Arabia

**Keywords:** Genome-wide analysis of gene expression, Genomics

## Abstract

Diatoms are the largest group of heterokont algae with more than 100,000 species. As one of the single-celled photosynthetic organisms that inhabit marine, aquatic and terrestrial ecosystems, diatoms contribute ~ 45% of global primary production. Despite their ubiquity and environmental significance, very few diatom plastid genomes (plastomes) have been sequenced and studied. This study explored patterns of nucleotide substitution rates of diatom plastids across the entire suite of plastome protein-coding genes for 40 taxa representing the major clades. The highest substitution rate was lineage-specific within the araphid 2 taxon *Astrosyne radiata* and radial 2 taxon *Proboscia* sp. Rate heterogeneity was also evident in different functional classes and individual genes. Similar to land plants, proteins genes involved in photosynthetic metabolism have lower synonymous and nonsynonymous substitutions rates than those involved in transcription and translation. Significant positive correlations were identified between substitution rates and measures of genomic rearrangements, including indels and inversions, which is a similar result to what was found in legume plants. This work advances the understanding of the molecular evolution of diatom plastomes and provides a foundation for future studies.

## Introduction

Diatoms are photosynthetic, unicellular eukaryotes of the heterokont algal lineage. Two hundred fifty million years ago, diatom plastids were derived from a secondary endosymbiotic event, in which a non-photosynthetic eukaryote phagocytized a red alga^1^. Diatoms have since colonized freshwater, marine and terrestrial habitats contributing ~ 45% of global primary production^2–4^ and as much as 20% of global carbon fixation via photosynthesis^5, 6^.

Despite their ubiquity and the environmental significance of diatom photosynthesis, very few diatom plastid genomes (plastomes) have been sequenced and studied. More than 2,900 plant species with plastomes were represented in the public databases based on searches in the NCBI on the February 4, 2019, but just 40 diatom taxa have been sequenced thus far^7^. The study of the sequences of plastomes can potentially reveal novel insights on relationships between monophyletic diatom lineages^7–11^. Researchers have also found support for the theory of shared ancestry between diatoms and rhodophytes^12^. Furthermore, the availability of plastomes has enabled exploration of the variation in structure and gene content across orders, genera and species^7, 9, 10, 13–16^.

Within the diatom cytoplasm, there are numerous or singular plastids of variable shapes^17, 18^. Four previously examined diatom species showed each of their plastids contained a single nucleoid^19^, which comprises copies of the plastome monomer or unit-genome, RNA and proteins^20^. Diatom plastid genes are densely arrayed on both strands of the unit-genome, which represent one full complement of the gene space and intergenic regions. The plastome of an individual may include many copies of the unit-genome by repeating this complement pattern many times. Although this unit is often diagrammed as a circular molecule, the plastome more likely contains a collection of circular, linear and linear-branched molecules that each comprises two to many copies of the monomer^21^. All diatom plastomes sequenced to date include a large inverted repeat (IR) separated by large and small single-copy regions (LSC and SSC, respectively). Apart from the typical quadripartite structure, an extensive range of gene order arrangements, gains and losses of genes are exhibited in the diatom plastomes^9, 10^. Gene order changes not only arise through gene duplication by IR expansion, but also via inversions and insertions and deletions (indels) in both IR and SC regions.

Calculation of synonymous (*dS*) and nonsynonymous (*dN*) nucleotide substitution rates across individual genes and their functional groups between lineages provide insights into the plastome evolution^22^. In previous studies, genes encoding subunits that are integral to photosynthesis, such as cytochrome b_6f._ complex (PET) and photosystems I and II (PSA and PSB) have lower rates of nucleotide substitution than other functional groups in angiosperms and conifers^23–26^. Accelerated substitution rates have been detected in ribosomal protein (RPL and RPS) genes and RNA polymerase (RPO) genes^24, 26–30^. Besides differences in substitution rates, variation relative to genomic features such as rearrangements in gene order can also shape plastome evolution. Previous studies have identified a significant positive correlation between rates of nucleotide substitution and gene order changes in angiosperm plastid genomes^30, 31^, bacterial genomes^32^ and arthropod mitochondrial genomes^33, 34^.

In a previous study by Schwarz et al.^26^, both nonsynonymous and synonymous substitution rates were negatively correlated with plastome sizes and rearrangements such as the number of inversions and indels. The focus of these investigations was on three of the six subfamilies of flowering plant family Fabaceae. One of the subfamilies, papilionoids, has a wide diversity of plastome rearrangements including the loss of inverted repeats (IR) in one clade and relatively smaller plastomes than the other subfamilies. This study also found that genes in the IR show three to fourfold reduction in substitution rates compared to SC regions. Genes that used to be in IR showed accelerated rates compared to genes retained in the IR. A negative correlation between substitution rates and cupressophyte plastid DNA genome size has also been reported in conifers^37^.

Our hypothesis is that the relationship of nucleotide substitution rates between plastid genes and plastome size and architecture such as inversions, indels, and IR in diatoms are similar to what was observed with legumes and conifers. If correct, this reflects a fundamental aspect of how diatoms evolve. To date, no study has investigated the nucleotide substitution rates of all shared plastome protein-coding genes in diatoms. The present study explored the patterns of plastid nucleotide substitution rates across the entire suite of 103 shared genes for 40 species of diatoms. Correlations between plastome substitution rates and genome features, including plastome size, number of indels and genome rearrangement were examined. This work advances the current understanding of the molecular evolution of diatom plastomes.

## Results

### Phylogenetic relationships and branch lengths

**Figure 1:**
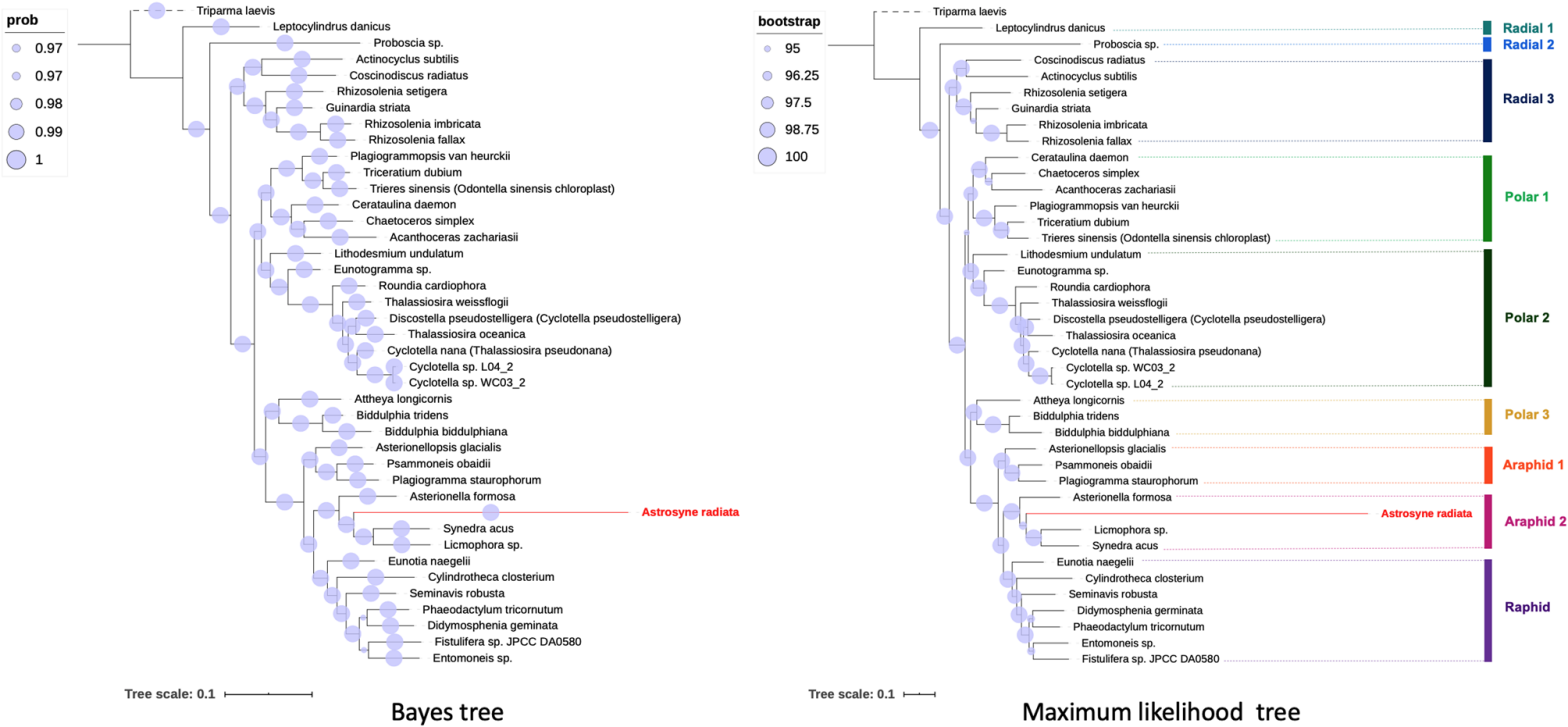
Bayes and maximum likelihood (ML) trees are constructed using 103 concatenated protein-coding gene sequences from each diatom. There is strong bootstrap support for all branches (> 97%) in the ML tree. *Astrosyne radiata* has an unusually long branch length in both Bayes and ML trees. 40 species are grouped into nine morphological categories. Note that the outgroup, *Triparma laevis*, is not included into the nine categories.

Phylogenetic analysis of 40 previously published diatom plastomes (Table S1) for the concatenated 103 gene data set (Table S2) generated Bayes and maximum likelihood (ML) trees with robust support of > 0.97 posterior probabilities and > 95% bootstraps for most of the branches, respectively (Fig. 1). The radial centrics of the Coscinodiscophyceae (radial 1, 2 and 3) formed a basal grade. The Mediophyceae including bi-polar and multi-polar diatoms plus the Thalassiosirales are paraphyletic and contained in three clades (polar 1, 2 and 3). Araphid 1 was sister to araphid 2, and phylogenetically close to another group, raphids. Raphid pennate diatoms were monophyletic. Within araphid 2, *Astrosyne radiata* showed an extremely long branch in both Bayes and ML trees (Fig. 1).

### Substitution rates in individual genes and functional groups

**Figure 2:**
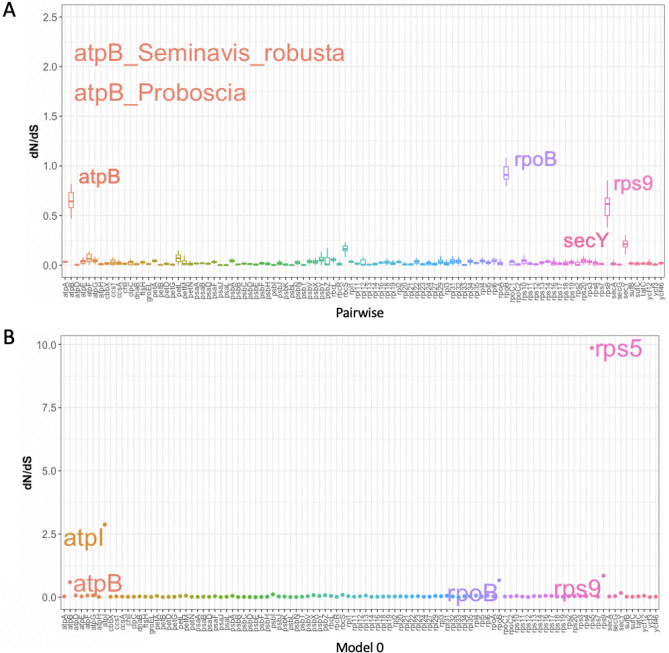
Distribution of *dN/dS* values for individual genes across all diatoms. Two types of *dN/dS* methods were used; (**A**) one based on pairwise *dN/dS* rates against the outgroup, *Triparma laevis*, and (**B**) the other method was based on PAML model 0 that produced a single *dN/dS* value for each orthologous set. The orthologous set has their gene names arranged in alphabetical order from left to right. Genes with higher *dN/dS* rates are indicated on the plot. In the pairwise model, the ratio was visualised by boxplot with each boxplot consisting of 40 species *dN/dS* values. *rps5* and *atpI* were not shown on the pairwise *dN/dS* boxplots because these are special cases subjected to CODEML model 8 vs 7 comparison in Table 1.

**Figure 3:**
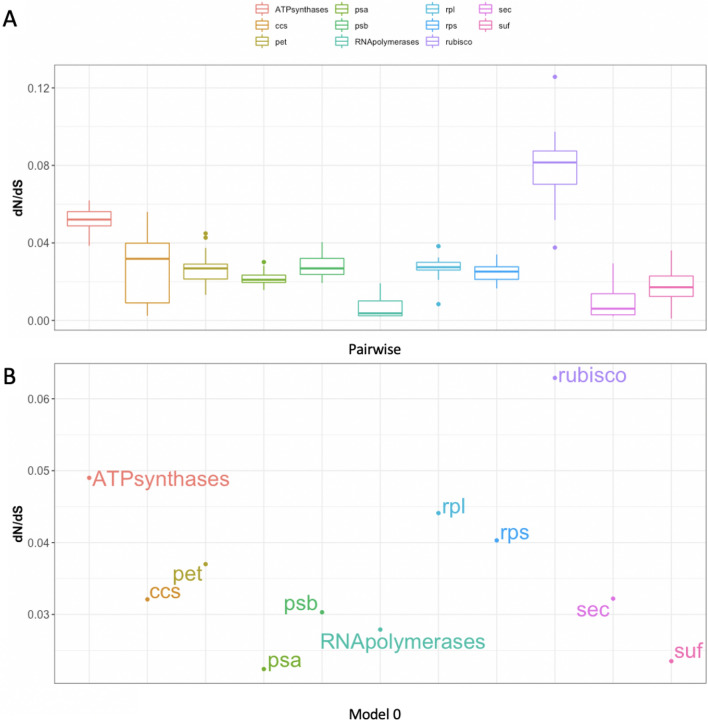
Distribution of *dN/dS* values for functional groups across all diatoms. The same pairwise and PAML model 0 *dN/dS* methods as described in the legend of Fig. 2 were used to calculate *dN/dS* values. The difference is functional groups consist of a set of genes. Table S2 shows how the genes were grouped into the 11 categories.

Estimations of *dN, dS* (Fig S1) and *dN*/*dS* (ω) (Figs. 2, 3) values were done for individual genes and different functional classes (Tables S1, S2). Two methods including pairwise and CODEML model 0 were used to estimate the nucleotide substitution rates. The *dN*/*dS* values were more widely distributed for individual genes (Fig. 2), whereas the ratio was limited to 0 to 0.13 for different functional classes (Fig. 3). Two genes, *rps*5 and *atp*I, have high *dN*/*dS* values > 1 and were subjected to positive selection analyses using CODEML model 8 versus 7. A total of 63 and 64 positively selected sites were detected in *rps*5 and *atp*I, respectively (Table 1). Four genes *atp*B, *rpo*B, *rps*9 and *sec*Y had higher *dN*/*dS* values, which suggested accelerated evolution of these genes. In particular, *atp*B in *Proboscia sp.* and *Seminavis robusta* showed *dN/dS* values of 1.75 and 2.15, respectively. When grouping genes into functional groups, all had *dN/dS* values close to 0 with the RubisCo subunit having median *dN/dS* values of only 0.08 (Fig. 3A), which suggested some genes with rapid evolution have their effects masked by other genes in the same functional group with purifying selection.

**Table 1:** CODEML model 8 vs 7 comparison for *rps5* and *atpI* and positively selected sites.

Gene	Model	Log-likelihood	2Δ (in L)	p-value	Positively selected sites	Tree length
rps5	M7	− 6,492.11				
	M8	− 6,275.91	432.4	1.28e–94	3 T, 7 K , 8 T, 9 N, 10R, 11I, 14C, 15 N, 24S, 25 N, 28 N, 29 W, 31 W, 33 R, 34 N, 35 W, 36 N, 37C, 40C, 42F, 45 K, 48S, 50 N, 51S, 52S, 53Y, 54 N, 55I, 57S, 58 T, 59C, 60C, 61L, 63Y, 64Y, 65 T, 66C, 67Y, 70R, 71G, 74R, 75 W, 76C, 77S, 78 N, 79S, 80I, 82C, 83R, 84Y, 87 N, 92I, 97S, 98C, 99 N, 100 W, 101F, 102I, 104 N, 105I, 107 K, 108 P, 109S	14.81
atpI	M7	− 9,013.33				
	M8	− 8,757.67	511.32	9.30e−112	1 K, 10L, 11 V, 13Y, 15 V, 18Y, 21Q, 22E, 23L, 24 K, 25I, 27L, 28 M, 30G, 36L, 37H, 38I, 39L, 41L, 43I, 48F, 53Y, 54H, 55I, 56 V, 57L, 63 V, 65I, 67 V, 68Q, 69F, 70L, 75Q, 77 V, 79Q, 80L, 81Q, 82L, 86L, 87L, 89R, 92P, 101D, 102 V, 104L, 107 M, 108L, 109 T, 110Q, 123L, 132, 133E, 136P, 139L, 140L, 142R, 144Y, 150Y, 151D, 154P, 163L, 164P, 166H, 170H	10.92

Bayes Empirical Bayes (BEB) was used to calculate posterior probabilities and only those with Prob(ω > 1) > 0.99 were shown. The p-value was calculated with degree of freedom equals to 2.

### Gene order in diatoms

Gene order analysis using MAUVE revealed substantial rearrangements of blocks of sequences in the 40 diatom species (Table 2). Only 14 species had identical gene order shared with at least one other species.

**Table 2:** Local collinear blocks (LCBs) for each of the 40 diatom plastomes identified by Mauve.

Species	Gene collinear block order
*Leptocylindrus danicus*	27 26 − 28 − 11 − 14 − 12 − 22 − 18 − 17 − 5 − 6 − 7 − 8 − 9 − 10 1 2 3 4 − 16 − 15 − 23 21 20 19 13 25 24 42 39 33 34 35 36 37 32 − 31 − 30 − 29 − 38 − 40 − 41
*Probosica *sp.	− 1 2 3 4 − 5 − 6 − 7 − 8 − 9 − 10 11 − 12 − 13 − 14 15 16 17 18 − 19 − 20 − 21 22 − 23 − 24 25 − 26 − 27 − 28 29 30 31 − 32 33 34 35 36 37 − 38 − 39 − 40 − 41 − 42
^*a*^*Actinocyclus subtilis*	2 3 4 − 13 − 19 − 20 − 21 − 1 − 11 − 14 − 12 − 22 − 18 − 17 − 5 − 6 − 7 − 8 − 9 − 10 23 15 16 28 27 26 25 24 42 41 40 39 38 29 30 31 33 34 35 36 37 32
^*a*^*Coscinodiscus radiatus*	2 3 4 − 13 − 19 − 20 − 21 − 1 − 11 − 14 − 12 − 22 − 18 − 17 − 5 − 6 − 7 − 8 − 9 − 10 23 15 16 28 27 26 25 24 42 41 40 39 38 29 30 31 33 34 35 36 37 32
*Rhizosolenia setigera*	2 3 4 − 13 − 19 − 20 − 21 − 1 − 11 − 14 − 12 − 22 − 18 − 17 − 5 − 6 − 7 − 8 − 9 − 10 23 15 16 28 27 26 25 24 42 41 − 32 − 37 − 36 − 35 − 34 − 33 29 30 31 − 38 − 39 − 40
*Guinardia striata*	2 3 4 − 13 23 15 16 28 27 26 10 9 8 7 6 5 17 18 22 12 14 11 1 21 20 19 − 41 − 42 − 24 − 25 − 32 − 37 − 36 − 35 − 34 − 33 29 30 31 − 38 − 39 − 40
*Rhizosolenia fallax*	2 3 4 − 13 − 19 − 20 − 21 − 1 − 11 − 14 − 12 − 22 − 18 − 17 − 5 − 6 − 7 − 8 − 9 − 10 − 26 − 27 − 28 − 16 − 15 − 23 25 24 42 41 − 32 − 37 − 36 − 35 − 34 − 33 29 30 31 − 38 − 39 − 40
*Rhizosolenia imbricate*	− 4 − 3 − 2 − 13 − 19 − 20 − 21 − 1 − 11 − 14 − 12 − 22 − 18 − 17 − 5 − 6 − 7 − 8 − 9 − 10 − 26 − 27 − 28 − 16 − 15 − 23 25 24 42 41 − 32 − 37 − 36 40 39 38 29 30 31 33 34 35
*Lithodesmium undulatum*	2 3 4 − 13 − 19 − 20 − 21 − 1 − 11 18 22 12 14 − 17 − 5 − 6 − 7 − 8 − 9 − 10 23 15 16 28 27 26 25 29 30 31 33 34 35 36 37 32 − 38 − 39 − 40 − 41 − 42 − 24
*Eunotogramma *sp.	22 12 14 11 16 28 27 26 2 3 4 − 13 − 19 − 20 − 21 − 18 − 17 − 5 − 6 − 7 − 8 − 9 − 10 15 − 23 1 25 24 42 41 40 39 38 30 31 33 34 35 36 37 32 − 29
^*b*^*Roundia cardiophora*	2 3 4 17 18 22 − 26 − 27 − 28 − 16 12 14 10 9 8 7 6 5 11 23 − 15 1 21 20 19 13 25 − 38 − 39 − 40 − 41 − 42 − 24 29 30 31 33 34 35 36 37 32
^*b*^*Thalassiosira weissflogii*	2 3 4 17 18 22 − 26 − 27 − 28 − 16 12 14 10 9 8 7 6 5 11 23 − 15 1 21 20 19 13 25 − 38 − 39 − 40 − 41 − 42 − 24 29 30 31 33 34 35 36 37 32
^*b*^*Discostella pseudostelligera*	2 3 4 17 18 22 − 26 − 27 − 28 − 16 12 14 10 9 8 7 6 5 11 23 − 15 1 21 20 19 13 25 − 38 − 39 − 40 − 41 − 42 − 24 29 30 31 33 34 35 36 37 32
*Thalassiosira oceania*	2 3 4 17 18 28 27 26 16 22 23 − 15 − 1 21 20 19 − 5 − 6 − 7 − 8 − 9 − 10 − 14 − 12 11 13 25 − 38 − 39 24 42 41 40 32 − 31 − 30 − 29 33 34 35 36 37
^*b*^*Cyclotella_nana*	2 3 4 17 18 22 − 26 − 27 − 28 − 16 12 14 10 9 8 7 6 5 11 23 − 15 1 21 20 19 13 25 − 38 − 39 − 40 − 41 − 42 − 24 29 30 31 33 34 35 36 37 32
^*c*^*Cyclotella *sp.* L04_2*	2 3 4 16 28 27 26 − 22 − 18 − 17 12 14 10 9 8 7 6 5 11 23 − 15 1 21 20 19 13 25 − 38 − 39 − 40 − 41 − 42 − 24 29 30 31 33 34 35 36 37 32
^*c*^*Cyclotella *sp.* WC03_2*	2 3 4 16 28 27 26 − 22 − 18 − 17 12 14 10 9 8 7 6 5 11 23 − 15 1 21 20 19 13 25 − 38 − 39 − 40 − 41 − 42 − 24 29 30 31 33 34 35 36 37 32
*Plagiogrammopsis van heurckii*	2 3 4 10 9 8 7 6 5 17 18 22 12 14 1 21 20 19 13 15 16 23 − 26 − 27 − 28 11 25 24 42 41 40 39 38 − 29 30 31 33 34 35 36 37 32
^*d*^*Trieres sinensis*	2 3 4 10 9 8 7 6 5 17 18 22 12 14 1 21 20 19 13 15 16 28 27 26 23 11 25 − 38 − 39 − 40 − 41 − 42 − 24 29 30 31 33 34 35 36 37 32
^*d*^*Triceratium dubium*	2 3 4 10 9 8 7 6 5 17 18 22 12 14 1 21 20 19 13 15 16 28 27 26 23 11 25 − 38 − 39 − 40 − 41 − 42 − 24 29 30 31 33 34 35 36 37 32
*Cerataulina daemon*	2 3 4 − 23 10 9 8 7 6 5 17 18 22 12 14 1 21 20 19 13 15 16 28 27 26 11 25 − 38 − 39 − 40 − 41 − 42 − 24 29 30 31 33 34 35 36 37 32
*Acanthoceras zachariasii*	10 9 8 2 3 4 7 6 5 17 18 22 12 14 1 21 20 19 13 15 16 28 27 26 23 11 25 29 30 31 33 34 35 36 37 32 24 42 41 40 39 38
*Chaetoceros simplex*	10 9 8 2 3 4 7 6 5 17 18 22 12 14 1 − 19 − 20 − 21 13 15 16 28 27 26 23 11 25 29 30 31 33 34 35 36 37 32 24 42 41 40 39 38
*Attheya logicornis*	2 3 4 13 − 19 − 20 − 21 23 15 16 1 28 27 26 22 12 14 − 18 − 17 − 5 − 6 − 7 − 8 − 9 − 10 11 25 − 39 − 40 − 41 − 42 − 24 29 30 31 33 34 35 36 37 32 38
^*e*^*Biddulphia tridens*	2 3 4 13 − 19 − 20 − 21 23 15 16 28 27 26 1 − 14 − 12 − 22 − 18 − 17 − 5 − 6 − 7 − 8 − 9 − 10 11 25 29 30 31 33 34 35 36 37 32 24 42 41 40 39 38
^*e*^*Biddulphia biddulphiana*	2 3 4 13 − 19 − 20 − 21 23 15 16 28 27 26 1 − 14 − 12 − 22 − 18 − 17 − 5 − 6 − 7 − 8 − 9 − 10 11 25 29 30 31 33 34 35 36 37 32 24 42 41 40 39 38
*Asterionellopsis glacialis*	2 3 4 13 − 23 − 1 21 20 19 15 16 − 11 − 14 − 12 − 22 − 18 − 17 10 9 8 7 6 5 − 26 − 27 − 28 25 − 38 33 34 35 36 37 − 39 − 40 − 41 − 42 − 24 − 31 − 30 − 29 32
*Plagiogramma staurophorum*	− 21 1 23 − 4 − 3 − 2 10 9 8 7 6 5 17 18 22 12 14 11 − 16 − 15 28 27 26 20 19 13 25 − 38 − 39 − 40 − 41 − 42 − 24 − 29 30 31 33 34 35 36 37 32
*Psammoneis obaidii*	7 6 5 2 3 4 − 13 − 19 − 20 − 21 1 23 15 16 − 11 17 18 22 12 14 − 26 − 27 − 28 10 9 8 25 − 38 32 29 30 31 33 34 35 36 37 24 42 41 40 39
*Asterionella formosa*	2 3 4 − 26 − 27 − 28 10 9 8 7 6 5 17 18 22 12 14 23 15 16 − 11 1 21 20 19 13 25 − 38 − 39 − 40 − 41 − 42 − 24 29 30 31 33 34 35 36 37 − 32
*Astrosyne radiata*	− 20 − 21 − 1 23 15 16 − 11 − 14 − 12 − 22 − 18 − 17 − 5 − 6 − 7 − 8 − 9 − 10 28 27 19 − 13 − 4 − 3 − 2 26 25 − 38 30 31 29 − 32 33 34 35 36 37 24 42 41 40 39
*Synedra acus*	2 3 4 − 13 − 19 − 20 − 21 1 23 15 16 − 11 − 14 − 12 − 22 − 18 − 17 − 5 − 6 − 7 − 8 − 9 − 10 28 27 26 25 − 38 − 39 − 40 − 41 − 42 − 24 29 30 31 33 34 35 36 37 32
*Licmorphora *sp.	− 4 − 3 − 2 − 26 − 27 − 28 10 9 8 7 6 5 17 18 22 12 14 11 − 16 − 15 − 23 − 1 21 20 19 13 25 − 38 − 39 − 40 − 41 − 42 − 24 29 30 31 − 32 − 37 − 36 − 35 − 34 − 33
*Eunotia naegelii*	2 3 4 − 13 − 19 − 20 − 21 − 1 23 15 16 − 11 − 14 − 12 − 22 − 18 − 17 − 5 − 6 − 7 − 8 − 9 − 10 − 26 − 27 − 28 25 − 38 − 39 − 40 − 41 − 42 − 24 29 30 31 33 34 35 36 37 32
*Cylindrotheca closterium*	− 2 − 23 9 8 7 6 5 17 18 22 12 14 11 − 16 − 15 − 13 − 19 − 20 − 21 1 − 26 − 27 − 28 − 10 25 − 32 33 34 35 36 37 29 30 31 24 42 41 40 39 38 − 4 − 3
*Seminavis robusta*	− 4 − 3 − 2 − 13 − 19 − 20 − 21 − 1 23 15 16 − 11 − 14 − 12 − 22 − 18 − 17 − 5 − 6 − 7 − 8 − 9 − 10 26 − 27 − 28 25 − 38 − 39 − 40 − 41 − 42 − 24 − 37 − 36 − 35 − 34 − 33 − 31 − 30 − 29 32
*Entomoneis *sp.	− 4 − 3 − 2 − 13 − 19 − 20 − 21 − 1 23 15 16 − 11 − 14 − 12 − 22 − 18 − 17 − 5 − 6 − 7 − 8 − 9 − 10 − 26 − 27 − 28 − 25 − 29 30 31 33 34 35 36 37 32 24 42 41 40 39 38
*Fistulifera sp JPCC DA0580*	− 4 − 3 − 2 − 13 − 19 − 20 − 21 − 1 23 15 16 − 26 − 27 − 28 10 9 8 7 6 5 17 18 22 12 14 11 25 − 38 − 39 − 40 − 41 − 42 − 24 29 30 31 33 34 35 36 37 32
^*f*^*Didymosphenia geminata*	− 4 − 3 − 2 − 13 − 19 − 20 − 21 − 1 23 15 16 − 11 − 14 − 12 − 22 − 18 − 17 − 5 − 6 − 7 − 8 − 9 − 10 − 26 − 27 − 28 25 − 38 − 39 − 40 − 41 − 42 − 24 29 30 31 33 34 35 36 37 32
^*f*^*Phaeodactylum tricornutum*	− 4 − 3 − 2 − 13 − 19 − 20 − 21 − 1 23 15 16 − 11 − 14 − 12 − 22 − 18 − 17 − 5 − 6 − 7 − 8 − 9 − 10 − 26 − 27 − 28 25 − 38 − 39 − 40 − 41 − 42 − 24 29 30 31 33 34 35 36 37 32

Negative numbers indicate an inversion in a given LCB. Only one IR was included in this analysis. The same gene order is highlighted with the same superscript letter before the species name.

### Correlation of substitution rates and plastome characteristics

**Figure 4:**
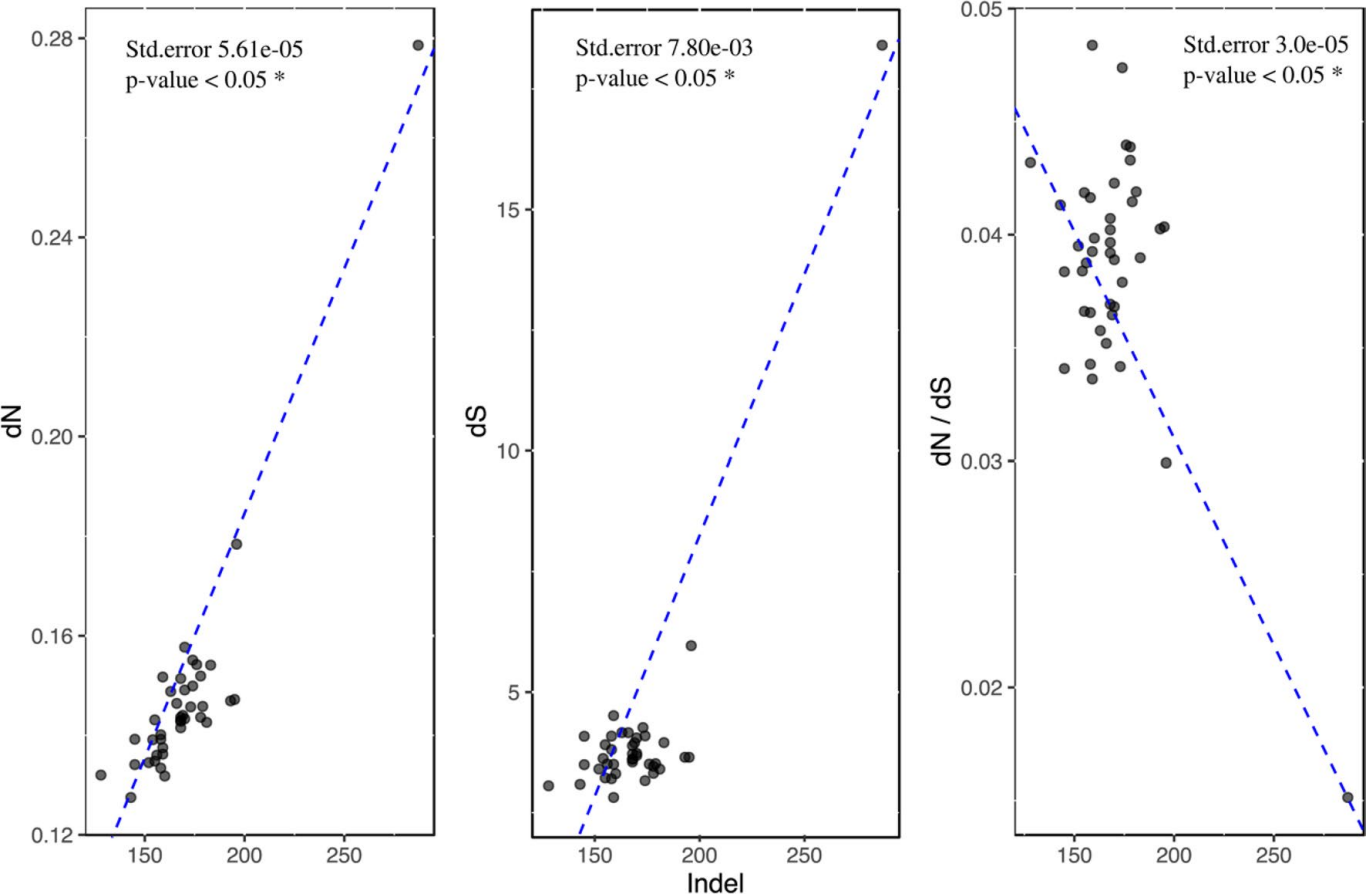
Relationship between the number of indels and substitution rates. Scatterplots were constructed and the regression line (dashed blue) and statistical values are shown. X-axis gives the number of indels in each species.

All correlations of the parameters of interest were visualised in Fig. S2. Significant correlation was observed between the number of indels and *dN*, *dS* and *dN/dS* (p < 0.05; Fig. 4). No significant correlation was found between the substitution rates and plastome size (Fig. S3). *Astrosyne radiata*, which has a relatively small plastome among diatoms, had the highest overall *dN* and *dS* (Fig. S3, Table S5)*.* Significant correlations were found between plastome size and the length of the inverted repeat (IR), and the length of the small/large single copy region (SSC, LSC respectively) (Fig. S4).

**Figure 5:**
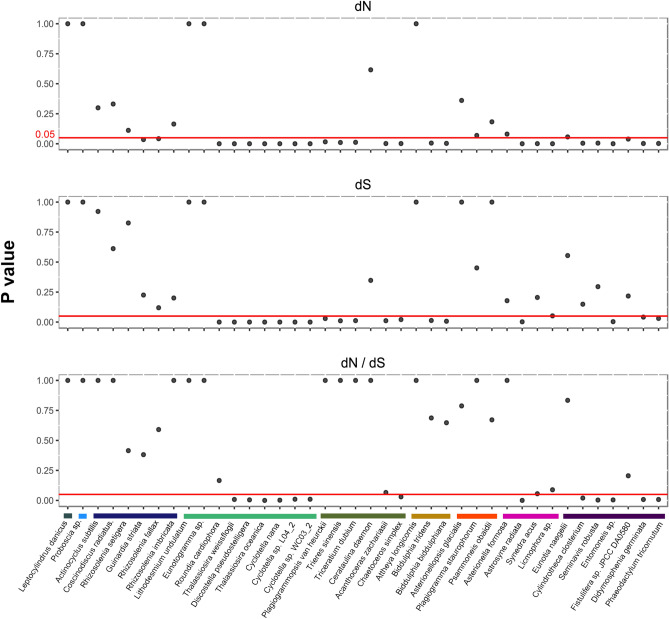
Pairwise correlation of substitution rate and plastome inversion distance in diatoms. 0.05 (red horizontal line) was used to assess the level of significance; P-values were plotted on the X-axis. Coloured bars indicate different clades of diatoms and correspond to Fig. 1. From left to right: radial 1, radial 2, radial 3, polar 1, polar 2, polar 3, araphid 1, araphid 2, raphid.

Correlation of pairwise substitution rates and inversion distance (Table S6) was tested in the 40 diatom plastomes. Significant correlation (p < 0.05) was found between *dN* and inversion distance in 25 out of 40 pairwise comparisons (Fig. 5; Table S6). Among the 40 plastomes, *dS* was significantly correlated with inversion distance in 18 pairwise comparisons, whereas the number of significant pairwise comparisons reduced to 13 for *dN/dS* values. The polar 1 group had the largest proportion of significant correlations between substitution rates and inversion distances. Seven of nine sampled taxa were significantly correlated in both *dN* and *dS*, and six of nine taxa were significantly correlated in *dN*/*dS*. *Astrosyne radiata*, which produced the longest branch in the diatom phylogeny (Fig. 1), also showed significant correlation of *dN* (p-value = 2.41e−06), *dS* (p-value = 2.23e−03), and *dN/dS* (p-value = 2.54e−03) with inversion distance (Fig. 5; Table S6).

## Discussion

Only limited studies have been performed using plastome protein-coding sequences from diatoms and not much is known about their molecular evolution. In this study, 103 plastid genes were examined across 40 diatom species, most of which were recently published by our group^7, 9, 10^. The ribosomal subunit and RNA polymerase genes have higher nucleotide substitution rates than other functional groups. Positive correlations are evident between *dN* and *dS* values and number of indels and inversion distances, which are proxies of genome rearrangements. Unlike the studies on legumes and conifers, we found no strong correlation between nucleotide substitution rates and diatom plastome size. The reason for the differences between diatoms and plants with respect to substitution rates may be attributed to fundamental differences in their genome content. Diatom plastomes are gene dense, with very little space dedicated to non-coding sequences and most are devoid of large repeat sequences^61^. However, the average diatom plastomes size is close to those of seed plants because they encode for more genes^7, 9, 10, 62^. Previous studies in diatoms also showed that variation in the unit-genome size is mainly due to expansion and contraction of the IR, gene loss and the introduction of foreign DNA of unknown origin^7, 9, 10^.

*Astrosyne radiata*, which is known to have undergone many gene loss events^7^, had the highest *dN* and *dS* but a relatively small plastome. This finding is in agreement with the legume plants^26, 37^. Perhaps species closely related to *A. radiata* will show the same negative correlation between substitution rates and plastome size but more sampling of related species is needed to confirm this observation. *Astrosyne radiata* is highly unusual from a morphological perspective, which perhaps explains its unusually long branch length in the phylogenetic tree. Although it is placed among araphid pennates, this diatom has elongated sternum and bilateral symmetry, and they have fully reverted to the ancestral radial symmetry (where all structures are rotationally arranged and symmetric around a single point in the centre) of diatoms in radial 3, such as *Coscinodiscus* and *Actinocyclus*.

Significant positive correlations were identified between substitution rates and two measures of genomic rearrangements, indels and inversions. This result is similar to legumes^26^ but not to conifers^37^. A recent study has also found significant correlations between branch length and gene order changes^17^ for two of the taxa in this study, *Astrosyne radiata* and *Proboscia* sp. This suggests that the evolutionary forces shaping the structural rearrangements between plants and diatoms are similar. Disruption of DNA repair, recombination and replication (DNA-RRR) systems has been suggested to cause highly elevated nucleotide substitution rates and genome rearrangements^24, 35^. A recent study revealed the potential correlation between *dN* rates of nuclear encoded DNA-RRR genes of plastomes and measures of plastome complexity in one angiosperm family^36^.

Like land plants, diatom plastid genes mainly fall into two general classes, those encoding proteins involved in photosynthetic metabolism (PSA, PSB, PET, ATP) and those with roles in transcription and translation (RPS, RPL, RPO). The finding that genes involved in photosynthesis had relatively lower overall substitution rates than genes in transcription-translation apparatus confirms that rate heterogeneity by functional class is a shared feature of diatoms and land plant plastomes.

Upon inspection of the protein alignments of the two positively selected genes, *rps*5 and *atp*I, we found that *Thalassiosira oceania* and *A. radiata* have very divergent sequences but were annotated with the correct gene symbols. The possibility of annotation errors leading to statistically significant positive selection cannot be discounted.

Gene essentiality is a widely studied factor in substitution rate variation, with the idea that essential genes are subject to stronger selective constraints than non-essential genes^45–47^. Several studies utilizing nuclear sequences have demonstrated that rates of nucleotide substitution are associated with gene expression levels where highly or more widely expressed genes evolve at slower rates in plants^48–50^ and animals^51–53^ supporting the notion that these genes may evolve under greater selective constraints. The slow rates of evolution in most of the genes examined in our study suggests they are essential genes.

In summary, positive correlations between nucleotide substitution rates and plastome rearrangements in both diatoms and legume plants motivate further studies to explore causal relationships between rates and plastome features. This will require expanded plastome sampling, both within and between diatom lineages. Future diatom studies should also consider the aspect of coevolution between nuclear and plastome genes, which has been done in several plant lineages^43, 44, 54, 55^.

## Methods

### Sequence alignment and phylogenetic analysis

Plastid protein-coding genes were extracted from all available complete diatom plastomes (40 taxa) together with the outgroup species *Triparma laevis* (Bolidophyceae) (Table S1). If similar sequences were annotated with the same gene names (i.e. isoforms) orthologs were selected using a phylogenetic tree-based approach^57^. Protein-coding genes were partitioned by functional category following Yu et al.^7^ The gene sequences were translated with the transeq function in EMBOSS v6.5.7^58^. Both gene and protein sequences were aligned with Multiple Alignment using Fast Fourier Transform (MAFFT v7.305)^38^. The aligned FASTA files of gene sequences were altered to PHYLIP format with ALTER v1.3.4^59^, and matched with the aligned protein sequences with PAL2NAL v14.1^60^. The Bayesian phylogenetic analysis was conducted under the GTR + G model using MrBayes v3.2.6^56^ with aligned protein sequences. The Markov chain Monte Carlo (mcmc) with default chain temperatures were run for 50,000 generations in two runs. The maximum likelihood trees were constructed with RAxML 7.2.9^39^, with the substitution model GTR + G and -f option. One thousand bootstrap replicates were performed to assess strength of support for clades. The maximum likelihood trees of individual genes and functional groups were then used as the constraint trees to estimate the substitution rates from individual-gene and functional-group levels, respectively.

### Nucleotide substitution rates

Nucleotide substitution rates (*dN* and *dS*) were estimated using the CODEML function implemented in PAML v4.8^40^. Gapped regions were excluded with the parameter “-nogap” flag in PAL2NAL to avoid spurious rate inference. Pairwise rates were calculated relative to the outgroup species *Triparma laevis* and estimated with the parameter runmode = − 2. All shared plastome genes (103) were concatenated for nucleotide substitution rate estimation and separate estimations were calculated on individual genes or concatenated sequences of genes in different functional groups as listed in Table S2. CODEML model 0 was also used to estimate *dN*/*dS* values at the level of individual genes and functional groups. For genes with *dN*/*dS* > 1 in model 0, these genes were tested further with CODEML model 7 (neutral) and model 8 (positive selection) to uncover potential positively selected sites using similar methodology as described previously^61^.

### Plastome features for correlation analyses

The number of indels for the concatenated 103 protein-coding genes was calculated using a custom Python script. *Triparma laevis* (Bolidophyceae) was used as a reference. Indels within aligned protein-coding regions were summed using a custom Python script resulting in a single value for each taxon; only intact genes were included (in-frame indels). Whole genome alignment among the 40 diatom species was performed using the ProgressiveMauve algorithm in Mauve v2.3.1^41^. The same IR copy (IRb) was removed from all plastomes. The locally collinear blocks (LCBs) identified by Mauve were numbered with positive or negative sign based on strand orientation to estimate genome rearrangement distance (Table S3). Pairwise inversion (IV) distances were estimated using Genome Rearrangements In Man and Mouse (GRIMM; Table S4)^42^. The feature ‘plastome size’ excludes one copy of the IR for each taxon.

### Correlation between substitution rates and genome characteristics

Pairwise *dN* and *dS* values were calculated for the 103 shared genes from each taxon relative to the outgroup *Triparma laevis*. Correlation of *dN* and *dS* with plastome size and indel number for each plastome was tested. Phylogenetic Generalized Least Squares was performed using the ape v5.4^62^ and nlme v3.1^63^ packages in R. The ML constraint tree was utilized with outgroup taxa pruned. The correlation between *dN* and *dS* with IV distance was tested using the Pearson test^64^. The resulting p-values were Bonferroni^65^ corrected using the built-in p.adjust function to account for the effect of multi-hypothesis testing.

## Supplementary information

Supplementary Information.

## Data Availability

The NCBI accession numbers of the diatoms used in this study: NC_024084.1, MG755791.1, MG755799.1, NC_024081.1, MG755793.1, MG755796.1, MG755802.1, NC_025311.1, NC_024085.1, MG755797.1, NC_025312.1, NC_025314.1, MG755804.1, NC_014808.1, NC_008589.1, KJ958480.1, KJ958481.1, MG755794.1, NC_001713.1, MG755801.1, NC_025313.1, MG755808.1, NC_025310.1, MG755798.1, MG755806.1, MG755805.1, NC_024080.1, MG755792.1, MG755803.1, NC_024079.1, MG755807.1, NC_016731.1, MG755795.1, NC_024928.1, NC_024082.1, MH356727.1, MG755800.1, NC_015403.1, NC_024083.1, NC_008588.1, NC_027746.1.
