# Supplementary material for: Nucleotide substitution rates of diatom plastid encoded protein genes are positively correlated with genome architecture
Source: Sci Rep. 2020 Sep 1;10:14358. doi: 10.1038/s41598-020-71473-1 (PMC7462845; doi:10.1038/s41598-020-71473-1)
Supplement: Supplementary file 1 — Supplementary Information. [file 41598_2020_71473_MOESM1_ESM.pdf]

Nucleotide substitution rates of diatom plastid encoded protein genes are positively correlated with genome architecture

Yan Ren[1], Mengjie Yu[2], Wai Yee Low[1], Tracey A Ruhlman[2], Nahid H Hajrah[3], Abdelfatteh El Omri[3], Mohammad K Alghamdi[4], Mumdooh J Sabir[5], Alawiah M. Alhebshi[3], Majid R Kamli[3], Jamal S.M. Sabir[3], Edward C. Theriot[2], Robert K Jansen[2#], Irfan A. Rather[3#]

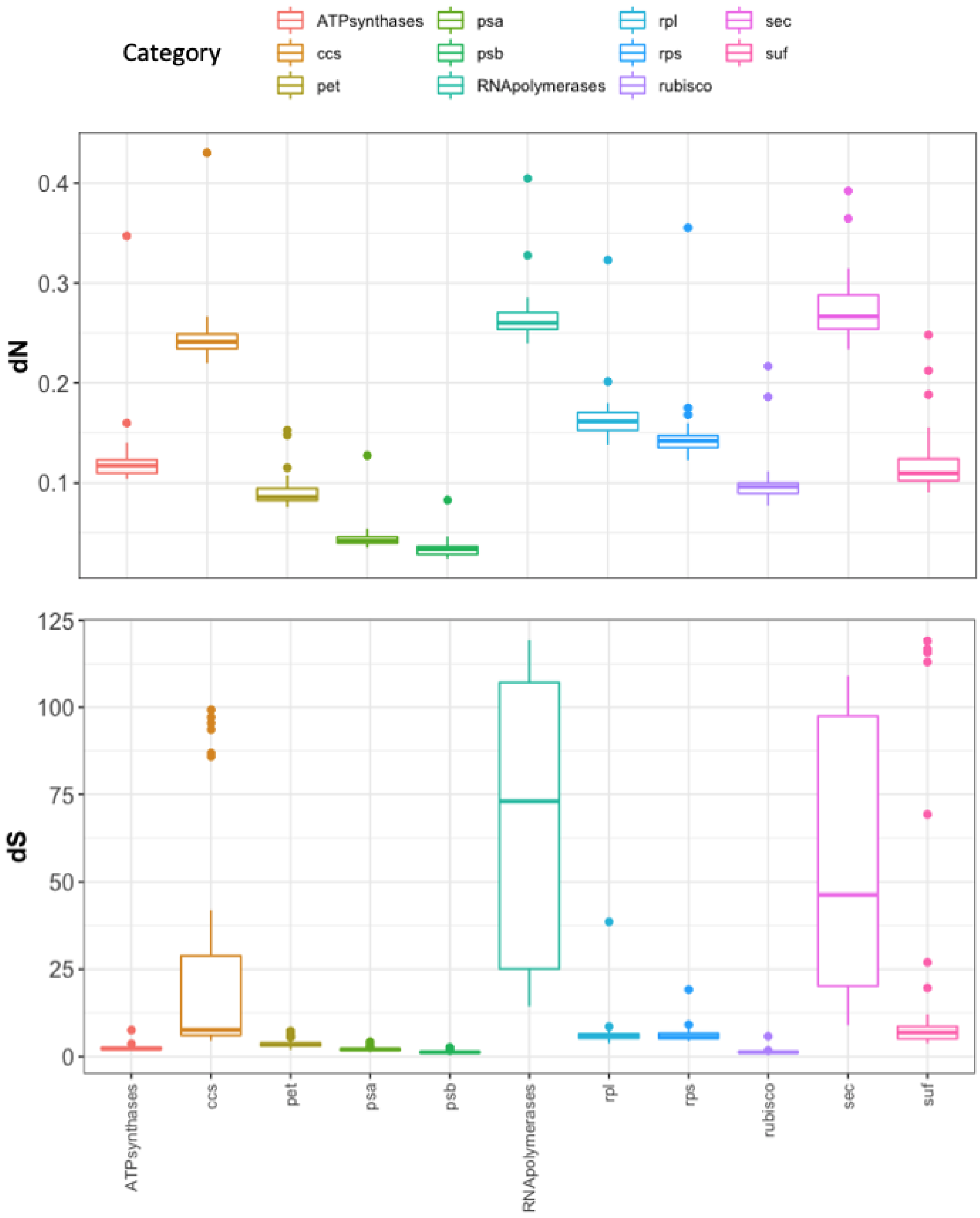

Figure S1. **Distribution of the nonsynonymous (*dN*) and synonymous (*dS*) substitution rates for functional groups of genes across all diatoms**. Genes are grouped as Table S2 to calculate the substitution rates for 11 categories. Genes in transcription and translation (*rps*, *rpl*, and RNA polymerase) have lower dN and dS rates than genes in photosynthetic metabolism (*psa*, *psb*, *pet*, and ATP).

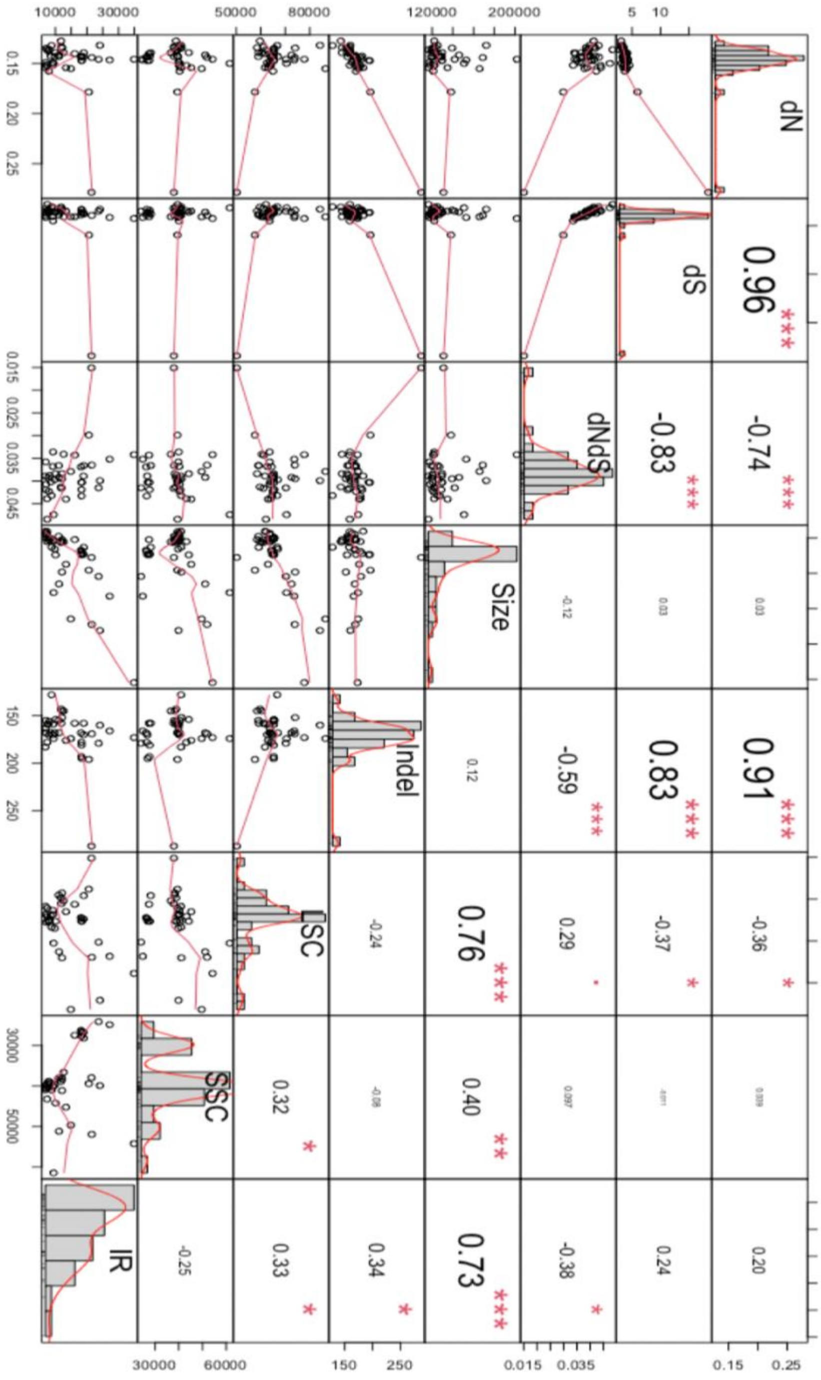

Figure S2. Pairwise correlation analysis of all the parameters in table S5. The parameters include the rates of *dN*, *dS* and *dN/dS*(ω), as well as different plastome features such as plastome size, Indel, LSC (large single-copy region of the plastome monomer), SSC (small single-copy region of the plastome monomer), IR (a single copy of the inverted repeat).

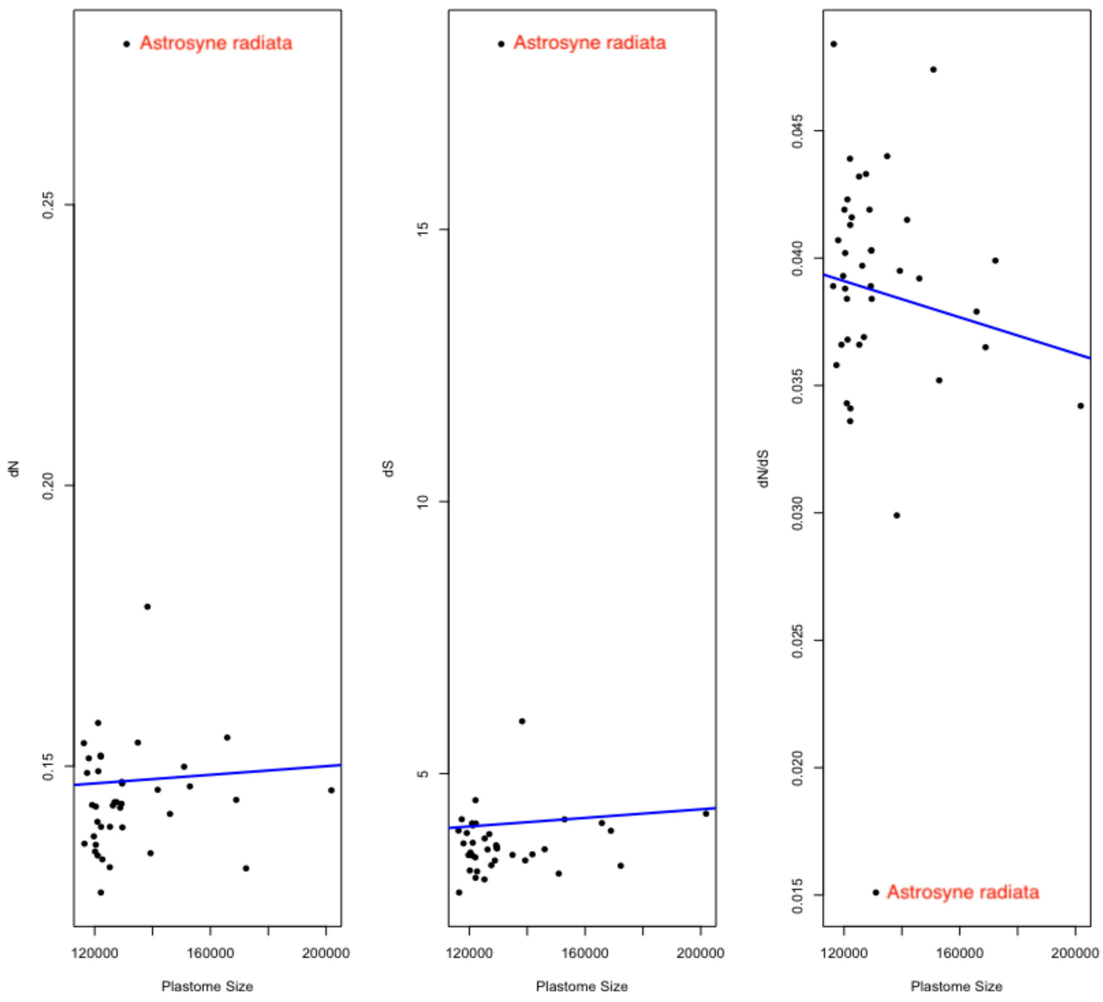

Figure S3. The correlation between substitution rates and plastome size. Each dot represents one species. *Astrosyne radiata* had the highest rates in both synonymous and nonsynonymous substitution, but the lowest ratio of *dN/dS*.

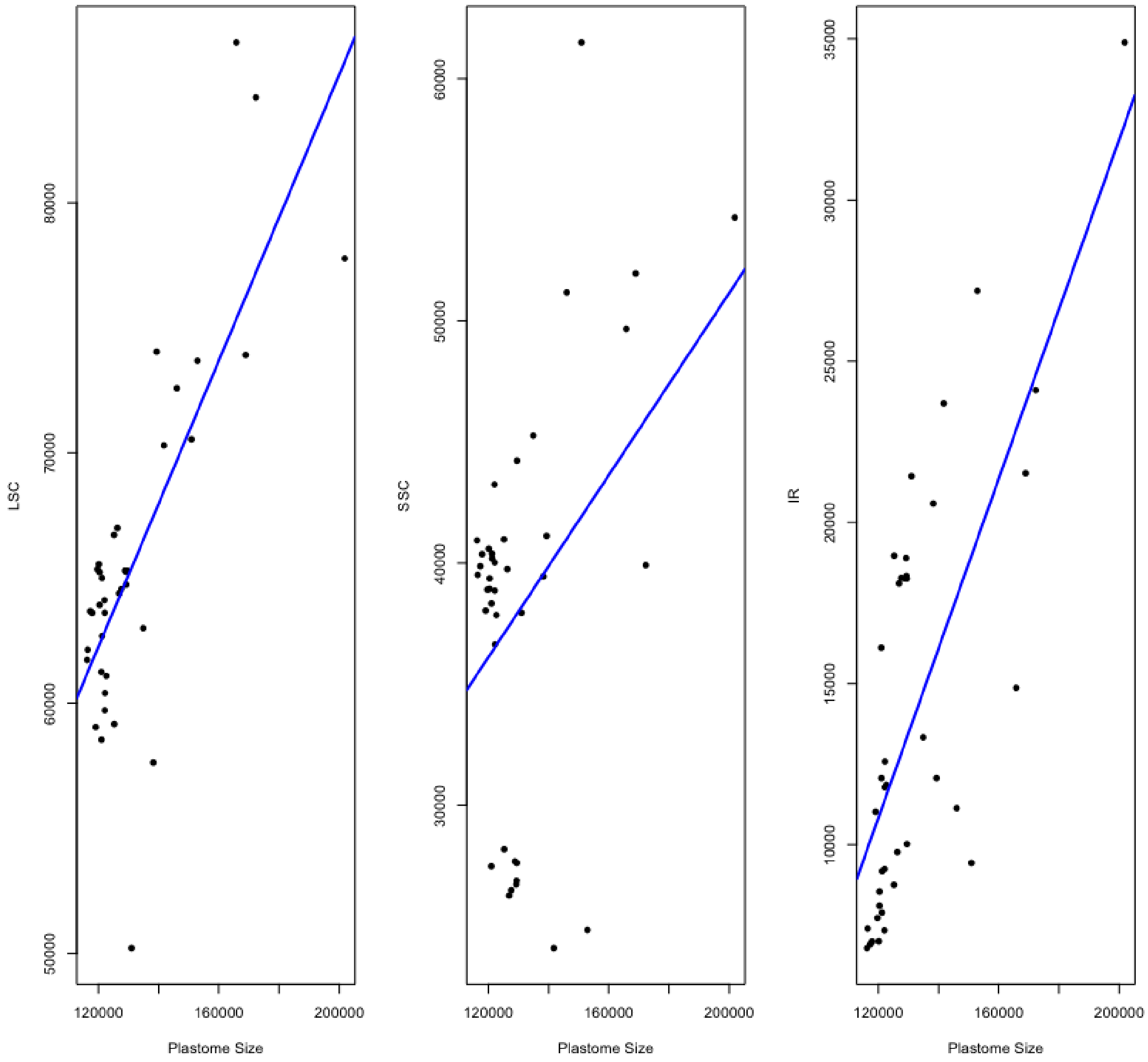

Figure S4. The potential correlation between the plastome size and different pattern features. The possible positive relationships were discovered between plastome size and other features, including small (SSC) and large (LSC) single copy region of the plastome monomer and inverted repeat (IR).

Table S1. Taxa included in diatom analyses and NCBI accession numbers. (*Triparma laevis* is the outgroup)

**Species**	**Accession Number**
Leptocylindrus danicus	NC_024084.1
Proboscia sp.	MG755791.1
Actinocyclus subtilis	MG755799.1
Coscinodiscus radiatus	NC_024081.1
Rhizosolenia setigera	MG755793.1
Guinardia striata	MG755796.1
Rhizosolenia fallax	MG755802.1
Rhizosolenia imbricata	NC_025311.1
Lithodesmium undulatum	NC_024085.1
Eunotogramma sp.	MG755797.1
Roundia cardiophora	NC_025312.1
Thalassiosira weissflogii	NC_025314.1
Discostella pseudostelligera (Cyclotella pseudostelligera)	MG755804.1
Thalassiosira oceanica	NC_014808.1
Cyclotella nana (Thalassiosira pseudonana)	NC_008589.1
Cyclotella sp. L04_2	KJ958480.1
Cyclotella sp. WC03_2	KJ958481.1
Plagiogrammopsis van heurckii	MG755794.1
Trieres sinensis (Odontella sinensis chloroplast)	NC_001713.1
Triceratium dubium	MG755801.1
Cerataulina daemon	NC_025313.1
Acanthoceras zachariasii	MG755808.1
Chaetoceros simplex	NC_025310.1
Attheya longicornis	MG755798.1
Biddulphia tridens	MG755806.1
Biddulphia biddulphiana	MG755805.1
Asterionellopsis glacialis	NC_024080.1
Plagiogramma staurophorum	MG755792.1
Psammoneis obaidii	MG755803.1
Asterionella formosa	NC_024079.1
Astrosyne radiata	MG755807.1
Synedra acus	NC_016731.1
Licmophora sp.	MG755795.1
Eunotia naegelii	NC_024928.1
Cylindrotheca closterium	NC_024082.1
Seminavis robusta	MH356727.1
Entomoneis sp.	MG755800.1
Fistulifera sp. JPCC DA0580	NC_015403.1

Didymosphenia geminata	NC_024083.1
Phaeodactylum tricornutum	NC_008588.1
Triparma laevis	NC_027746.1

Table S2. Plastid genes and functional groups included in rates analyses

**Category**	**Genes**
Photosystem I (PSA)	*psaA, psaB, psaD, psaF, psaJ, psaL*
Photosystem II (PSB)	*psbA, psbB, psbC, psbD, psbE, psbF, psbH, psbI , psbJ, psbK, psbL, psbN, psbT, psbV, psbX, psbY, psbZ*
Cytochrome B_6f complex (PET)	*petA, petB, petD, petG, petL, petM, petN*
ATP synthase	*atpA, atpB, atpD, atpE, atpF, atpG, atpH, atpI*
RubisCo subunit	*rbcL, rbcS, rbcR*
RNA polymerase (RPO)	*rpoA, rpoB, rpoC1, rpoC2*
Ribosomal proteins large subunit (RPL)	*rpl1, rpl2, rpl3, rpl4, rpl5, rpl6, rpl11, rpl12, rpl13, rpl14, rpl16, rpl18, rpl19, rpl20, rpl21, rpl22, rpl23, rpl24, rpl27, rpl29, rpl31, rpl32, rpl33, rpl34, rpl35*
Ribosomal proteins small subunit (RPS)	*rps2, rps3, rps4, rps5, rps7, rps9, rps10, rps11, rps12, rps13, rps14, rps16, rps17, rps18, rps19, rps20*
Cytochrome c biogenesis protein	*ccs1, ccsA*
Protein translocase subunit	*secA, secG, secY*
Fe-S cluster assembly protein	*sufB, sufC*
Other genes	*cbbX, chlI, clpC, dnaB, ftsH, groEL, tatC, ycf3, ycf12, ycf46*

Table S3. Pairwise inversion distance of 40 diatom plastomes inferred by Genome Rearrangements In Man and Mouse (GRIMM, http://www-cse.ucsd.edu/groups/bioinformatics/GRIMM.).

	Leptocylindrus danicus	Proboscia sp.	Actinocyclus subtilis	Coscinodiscus radiatus	Rhizosolenia setigera	Guinardia striata	Rhizosolenia fallax	Rhizosolenia imbricata	Lithodesmium undulatum	Eunotogramma sp.	Roundia cardiophora	Thalassiosira weissflogii	Discostella pseudostelligera	Thalassiosira oceanica	Cyclotella nana	Cyclotella sp L04_2	Cyclotella sp WC03_2	Plagiogrammopsis van heurckii	Trieres sinensis	Triceratium dubium
Leptocylindrus danicus	0	19	11	11	13	13	13	12	14	16	16	16	16	17	16	16	16	14	13	13
Probosica sp	19	0	16	16	17	19	18	18	17	18	19	19	19	19	19	18	18	18	17	17
Actinocyclus subtilis	11	16	0	0	2	5	3	4	3	8	9	9	9	15	9	9	9	7	6	6
Coscinodiscus radiatus	11	16	0	0	2	5	3	4	3	8	9	9	9	15	9	9	9	7	6	6
Rhizosolenia setigera	13	17	2	2	0	3	1	4	5	10	11	11	11	15	11	11	11	9	8	8
Guinardia striata	13	19	5	5	3	0	2	5	8	11	13	13	13	16	13	12	12	10	10	10
Rhizosolenia fallax	13	18	3	3	1	2	0	3	6	11	11	11	11	16	11	10	10	8	8	8
Rhizosolenia imbricata	12	18	4	4	4	5	3	0	7	12	12	12	12	19	12	11	11	9	9	9
Lithodesmium undulatum	14	17	3	3	5	8	6	7	0	9	12	12	12	15	12	12	12	10	9	9
Eunotogramma sp	16	18	8	8	10	11	11	12	9	0	13	13	13	18	13	13	13	10	11	11
Roundia cardiophora	16	19	9	9	11	13	11	12	12	13	0	0	0	10	0	1	1	10	8	8
Thalassiosira weissflogii	16	19	9	9	11	13	11	12	12	13	0	0	0	10	0	1	1	10	8	8
Discostella pseudostelligera	16	19	9	9	11	13	11	12	12	13	0	0	0	10	0	1	1	10	8	8
Thalassiosira oceania	17	19	15	15	15	16	16	19	15	18	10	10	10	0	10	11	11	16	14	14
Cyclotella nana	16	19	9	9	11	13	11	12	12	13	0	0	0	10	0	1	1	10	8	8
Cyclotella sp L04_2	16	18	9	9	11	12	10	11	12	13	1	1	1	11	1	0	0	11	8	8
Cyclotella sp WC03_2	16	18	9	9	11	12	10	11	12	13	1	1	1	11	1	0	0	11	8	8
Plagiogrammopsis van heurckii	14	18	7	7	9	10	8	9	10	10	10	10	10	16	10	11	11	0	4	4
Trieres sinensis	13	17	6	6	8	10	8	9	9	11	8	8	8	14	8	8	8	4	0	0
Triceratium dubium	13	17	6	6	8	10	8	9	9	11	8	8	8	14	8	8	8	4	0	0
Cerataulina daemon	13	17	4	4	6	9	7	8	7	12	7	7	7	15	7	6	6	5	2	2
Acanthoceras zachariasii	16	18	9	9	11	12	11	11	9	13	12	12	12	17	12	12	12	7	4	4
Chaetoceros simplex	17	18	10	10	12	13	12	12	10	14	13	13	13	17	13	13	13	8	5	5
Attheya logicornis	15	18	10	10	12	13	11	12	11	13	14	14	14	17	14	13	13	12	9	9
Biddulphia tridens	13	17	7	7	8	11	9	10	6	11	11	11	11	15	11	12	12	10	7	7
Biddulphia biddulphiana	13	17	7	7	8	11	9	10	6	11	11	11	11	15	11	12	12	10	7	7
Asterionellopsis glacialis	16	17	11	11	11	12	10	13	13	17	14	14	14	16	14	13	13	13	11	11
Plagiogramma staurophorum	12	17	10	10	12	13	11	12	12	13	11	11	11	17	11	10	10	9	9	9
Psammoneis obaidii	17	19	11	11	13	14	14	15	11	14	14	14	14	17	14	15	15	15	12	12
Asterionella formosa	13	17	6	6	8	11	9	10	8	12	8	8	8	15	8	8	8	9	7	7
Astrosyne radiata	18	20	11	11	12	14	12	14	13	14	16	16	16	20	16	16	16	15	13	13
Synedra acus	12	16	4	4	6	9	7	8	7	10	10	10	10	15	10	10	10	9	7	7
Licmorphora sp	10	16	7	7	9	10	10	9	9	11	11	11	11	14	11	11	11	11	9	9
Eunotia naegelii	11	16	4	4	6	7	5	6	7	10	9	9	9	15	9	8	8	7	5	5
Cylindrotheca closterium	15	18	11	11	11	13	11	12	12	15	14	14	14	18	14	13	13	13	11	11
Seminavis robusta	14	19	7	7	9	10	8	7	9	13	12	12	12	17	12	11	11	10	8	8
Entomoneis sp	14	19	8	8	10	10	9	8	8	11	12	12	12	17	12	11	11	9	10	10
Fistulifera sp JPCC DA0580	11	17	5	5	7	8	6	5	7	11	10	10	10	16	10	9	9	8	6	6
Didymosphenia germinata	12	17	5	5	7	8	6	5	7	11	10	10	10	16	10	9	9	8	6	6
Phaeodactylum tricornutum	12	17	5	5	7	8	6	5	7	11	10	10	10	16	10	9	9	8	6	6

	Cerataulina daemon	Acanthoceras zachariasii	Chaetoceros simplex	Attheya longicornis	Biddulphia tridens	Biddulphia biddulphiana	Asterionellopsis glacialis	Plagiogramma staurophorum	Psammoneis obaidii	Asterionella formosa	Astrosyne radiata	Synedra acus	Licmophora sp.	Eunotia naegelii	Cylindrotheca closterium	Seminavis robusta	Entomoneis sp.	Fistulifera sp JPCC DA0580	Didymosphenia germinata	Phaeodactylum tricornutum
Leptocylindrus danicus	13	16	17	15	13	13	16	12	17	13	18	12	10	11	15	14	14	11	12	12
Probosica sp	17	18	18	18	17	17	17	17	19	17	20	16	16	16	18	19	19	17	17	17
Actinocyclus subtilis	4	9	10	10	7	7	11	10	11	6	11	4	7	4	11	7	8	5	5	5
Coscinodiscus radiatus	4	9	10	10	7	7	11	10	11	6	11	4	7	4	11	7	8	5	5	5
Rhizosolenia setigera	6	11	12	12	8	8	11	12	13	8	12	6	9	6	11	9	10	7	7	7
Guinardia striata	9	12	13	13	11	11	12	13	14	11	14	9	10	7	13	10	10	8	8	8
Rhizosolenia fallax	7	11	12	11	9	9	10	11	14	9	12	7	10	5	11	8	9	6	6	6
Rhizosolenia imbricata	8	11	12	12	10	10	13	12	15	10	14	8	9	6	12	7	8	5	5	5
Lithodesmium undulatum	7	9	10	11	6	6	13	12	11	8	13	7	9	7	12	9	8	7	7	7
Eunotogramma sp	12	13	14	13	11	11	17	13	14	12	14	10	11	10	15	13	11	11	11	11
Roundia cardiophora	7	12	13	14	11	11	14	11	14	8	16	10	11	9	14	12	12	10	10	10
Thalassiosira weissflogii	7	12	13	14	11	11	14	11	14	8	16	10	11	9	14	12	12	10	10	10
Discostella pseudostelligera	7	12	13	14	11	11	14	11	14	8	16	10	11	9	14	12	12	10	10	10
Thalassiosira oceania	15	17	17	17	15	15	16	17	17	15	20	15	14	15	18	17	17	16	16	16
Cyclotella nana	7	12	13	14	11	11	14	11	14	8	16	10	11	9	14	12	12	10	10	10
Cyclotella sp L04_2	6	12	13	13	12	12	13	10	15	8	16	10	11	8	13	11	11	9	9	9
Cyclotella sp WC03_2	6	12	13	13	12	12	13	10	15	8	16	10	11	8	13	11	11	9	9	9
Plagiogrammopsis van heurckii	5	7	8	12	10	10	13	9	15	9	15	9	11	7	13	10	9	8	8	8
Trieres sinensis	2	4	5	9	7	7	11	9	12	7	13	7	9	5	11	8	10	6	6	6
Triceratium dubium	2	4	5	9	7	7	11	9	12	7	13	7	9	5	11	8	10	6	6	6
Cerataulina daemon	0	6	7	9	7	7	10	8	12	7	12	6	9	4	9	7	9	5	5	5
Acanthoceras zachariasii	6	0	1	11	7	7	15	12	13	11	17	11	12	9	12	11	9	9	9	9
Chaetoceros simplex	7	1	0	12	8	8	15	13	14	12	18	12	13	10	13	12	10	10	10	10
Attheya logicornis	9	11	12	0	5	5	12	13	12	11	12	9	12	8	13	11	11	9	9	9
Biddulphia tridens	7	7	8	5	0	0	11	12	12	9	13	8	11	7	12	10	8	8	8	8
Biddulphia biddulphiana	7	7	8	5	0	0	11	12	12	9	13	8	11	7	12	10	8	8	8	8
Asterionellopsis glacialis	10	15	15	12	11	11	0	10	11	11	11	7	10	7	10	8	12	10	8	8
Plagiogramma staurophorum	8	12	13	13	12	12	10	0	12	8	14	6	6	6	11	9	7	8	7	7
Psammoneis obaidii	12	13	14	12	12	12	11	12	0	9	11	7	8	9	14	10	11	10	10	10
Asterionella formosa	7	11	12	11	9	9	11	8	9	0	10	4	5	4	12	7	7	5	5	5
Astrosyne radiata	12	17	18	12	13	13	11	14	11	10	0	8	11	8	13	9	12	9	9	9
Synedra acus	6	11	12	9	8	8	7	6	7	4	8	0	3	2	8	5	7	3	3	3
Licmorphora sp	9	12	13	12	11	11	10	6	8	5	11	3	0	5	9	6	8	4	4	4
Eunotia naegelii	4	9	10	8	7	7	7	6	9	4	8	2	5	0	9	3	5	3	1	1
Cylindrotheca closterium	9	12	13	13	12	12	10	11	14	12	13	8	9	9	0	10	10	9	8	8
Seminavis robusta	7	11	12	11	10	10	8	9	10	7	9	5	6	3	10	0	6	4	2	2
Entomoneis sp	9	9	10	11	8	8	12	7	11	7	12	7	8	5	10	6	0	6	4	4
Fistulifera sp JPCC DA0580	5	9	10	9	8	8	10	8	10	5	9	3	4	3	9	4	6	0	2	2
Didymosphenia germinata	5	9	10	9	8	8	8	7	10	5	9	3	4	1	8	2	4	2	0	0
Phaeodactylum tricornutum	5	9	10	9	8	8	8	7	10	5	9	3	4	1	8	2	4	2	0	0

Table S4. Nucleotide substitution rates and plastome features included in correlation analyses.

Species	*dN*	*dS*	*dN/dS*(ω)	Size	Indel	LSC	SSC	IR	Clade
Leptocylindrus danicus	0.1320	3.0563	0.0432	125213	128	66724	40981	8754	radial1
Proboscia sp.	0.1784	5.9626	0.0299	138249	196	57631	39450	20584	radial2
Actinocyclus subtilis	0.1431	3.9087	0.0366	119120	155	59040	38042	11019	radial3
Coscinodiscus radiatus	0.1392	4.0845	0.0341	122213	145	60402	36643	12584	radial3
Rhizosolenia setigera	0.1341	3.4955	0.0384	121011	145	58541	38332	12069	radial3
Guinardia striata	0.1275	3.0861	0.0413	122145	143	59711	38870	11782	radial3
Rhizosolenia fallax	0.1392	3.8077	0.0366	125283	158	59165	28184	18967	radial3
Rhizosolenia imbricata	0.1401	4.0877	0.0343	120956	158	61244	27482	16115	radial3
Lithodesmium undulatum	0.1334	3.2036	0.0416	122660	158	61086	37854	11860	polar1
Eunotogramma sp.	0.1318	3.3076	0.0399	172317	160	84201	39912	24102	polar1
Roundia cardiophora	0.1436	3.8890	0.0369	126871	168	64387	26274	18105	polar1
Thalassiosira weissflogii	0.1436	3.3167	0.0433	127601	178	64555	26494	18276	polar1
Discostella pseudostelligera	0.1433	3.6842	0.0389	129261	170	64734	26735	18896	polar1
Thalassiosira oceanica	0.1458	3.5168	0.0415	141790	179	70298	24106	23693	polar1
Cyclotella nana	0.1426	3.4036	0.0419	128814	181	65292	27684	18261	polar1
Cyclotella sp. L04_2	0.1472	3.6482	0.0403	129400	195	65250	26889	18338	polar1
Cyclotella sp. WC03_2	0.1469	3.6491	0.0403	129498	193	65268	27620	18256	polar1
Plagiogrammopsis van heurckii	0.1345	3.4051	0.0395	139305	152	74042	41125	12069	polar1
Trieres sinensis	0.1375	3.5028	0.0393	119704	159	65346	38908	7725	polar1
Triceratium dubium	0.1360	3.5093	0.0388	120381	156	65233	38936	8106	polar1
Cerataulina daemon	0.1348	3.2203	0.0419	120144	155	65546	40590	7004	polar2
Acanthoceras zachariasii	0.1428	3.5508	0.0402	120392	168	63924	39368	8550	polar2
Chaetoceros simplex	0.1362	2.8153	0.0484	116459	159	62136	39517	7403	polar2
Attheya longicornis	0.1391	3.6231	0.0384	129565	154	65290	44231	10022	polar3
Biddulphia tridens	0.1430	3.6062	0.0397	126295	168	66995	39752	9774	polar3
Biddulphia biddulphiana	0.1517	4.5121	0.0336	122128	159	63612	40024	9246	polar3

Asterionellopsis glacialis	0.1415	3.6106	0.0392	146024	168	72585	51181	11129	araphid1
Plagiogramma staurophorum	0.1457	4.2639	0.0342	201816	173	77767	54273	34888	araphid1
Psammoneis obaidii	0.144	3.9499	0.0365	168922	169	73911	51965	21523	araphid1
Asterionella formosa	0.1491	4.0496	0.0368	121238	170	62681	40193	9182	araphid2
Astrosyne radiata	0.2786	18.4078	0.0151	131032	287	50213	37953	21433	araphid2
Synedra acus	0.1541	3.9532	0.0389	116251	183	61724	40937	6795	araphid2
Licmophora sp.	0.1577	3.73	0.0423	121184	170	64999	40389	7898	araphid2
Eunotia naegelii	0.1464	4.1596	0.0352	152906	166	73679	24857	27185	raphid
Cylindrotheca closterium	0.1551	4.0927	0.0379	165809	174	86398	49671	14870	raphid
Seminavis robusta	0.1499	3.1636	0.0474	150905	174	70540	61497	9434	raphid
Entomoneis sp.	0.1519	3.4616	0.0439	122056	178	64114	43246	7348	raphid
Fistulifera sp. JPCC DA0580	0.1542	3.5073	0.0440	134918	176	62994	45264	13330	raphid
Didymosphenia geminata	0.1514	3.7183	0.0407	117972	168	63610	40370	6996	raphid
Phaeodactylum tricornutum	0.1488	4.1613	0.0358	117369	163	63674	39871	6912	raphid

dN: nonsynonymous substitution rate
dS: synonymous substation rate
dN/dS(ω): ratio of substitution rates
Size: total plastome size in nucleotides (nt)
Indel: in-frame (coding region) insertions and deletions
LSC: large single copy region of the plastome monomer (nt)
SSC: small single copy region of the plastome monomer (nt)
IR: a single copy of the inverted repeat (IR) (nt)

Table S5. Correlation coefficient and adjusted P-values for correlation between substitution rates and plastome rearrangement measured by inversion distance. Significant p-values are highlighted in red font.

**Species**	***dN* Cor**	***dN* Adjusted P value**	***dS* Cor**	***dS* Adjusted P value**	***dN/dS* (ω) *Cor***	***dN/dS*(ω) Adjusted P value**	**Clade**
Leptocylindrus danicus	0.249362463	1	0.274406108	1	-0.0947601	1	radial1
***Proboscia* sp.**	0.281901059	1	0.209321483	1	0.10195213	1	radial2
Actinocyclus subtilis	0.421786386	0.299	0.363209145	0.922	0.25090632	1	radial3
Coscinodiscus radiatus	0.416940345	0.331	0.38572864	0.612	0.24939738	1	radial3
Rhizosolenia setigera	0.466018696	0.112	0.369400225	0.826	0.40581409	0.415	radial3
Guinardia striata	0.511819208	3.47×10^{-2}	0.43514724	0.225	0.41001054	0.381	radial3
Rhizosolenia fallax	0.504714175	4.20×10^{-2}	0.463100951	0.120	0.38761555	0.591	radial3
Rhizosolenia imbricate	0.449402086	1.64×10^{-1}	0.440441621	0.201	0.25435017	1	radial3
Lithodesmium undulatum	0.173043419	1	0.163370113	1	-0.1890741	1	polar1
***Eunotogramma* sp.**	0.142648235	1	0.161402882	1	-0.1539923	1	polar1
Roundia cardiophora	0.674043907	1.02×10^{-4}	0.689209635	4.90×10^{-5}	0.44892442	0.166	polar1
Thalassiosira weissflogii	0.697095747	3.28×10^{-5}	0.695149186	3.63×10^{-5}	0.56102947	8.12×10^{-3}	polar1
Discostella pseudostelligera	0.696753636	3.34×10^{-5}	0.699876785	2.84×10^{-5}	0.57780361	4.69×10^{-3}	polar1
Thalassiosira oceanica	0.775774078	2.64×10^{-7}	0.778485249	2.16E-07	0.67354051	1.05×10^{-4}	polar1
Cyclotella nana	0.710748719	1.59×10^{-5}	0.713458405	1.37×10^{-5}	0.60046134	2.13×10^{-3}	polar1
***Cyclotella* sp.L04_2**	0.702493306	2.48×10^{-5}	0.714705524	1.28×10^{-5}	0.55446278	9.99×10^{-3}	polar1
***Cyclotella* sp.WC03_2**	0.702450644	2.48×10^{-5}	0.714486026	1.30×10^{-5}	0.55495347	9.83×10^{-3}	polar1
Plagiogrammopsis van heurckii	0.539178885	1.59×10^{-2}	0.517654516	2.95×10^{-2}	0.29125934	1	polar1
Trieres sinensis	0.55324712	1.04×10^{-2}	0.55223179	1.07×10^{-2}	0.35044535	1	polar1
Triceratium dubium	0.54893446	1.18×10^{-2}	0.548404246	1.20×10^{-2}	0.34965209	1	polar1
Cerataulina daemon	0.385386993	0.616	0.414586181	0.347	0.12967364	1	polar2
Acanthoceras zachariasii	0.607803552	1.62×10^{-3}	0.549880351	1.15×10^{-2}	0.48724442	6.62×10^{-2}	polar2
Chaetoceros simplex	0.607150507	1.67×10^{-3}	0.529659953	2.10×10^{-2}	0.51703089	3.04×10^{-2}	polar2
Attheya longicornis	0.324697507	1	0.330594406	1	0.28321125	1	polar3
Biddulphia tridens	0.573014743	5.50×10^{-3}	0.545305193	1.32×10^{-2}	0.37951437	0.687	polar3
Biddulphia biddulphiana	0.590314317	3.05×10^{-3}	0.565746504	6.98×10^{-3}	0.38273125	0.647	polar3
Asterionellopsis glacialis	0.41273536	0.361	0.250482164	1	0.37204151	0.788	araphid1
Plagiogramma staurophorum	0.48560559	6.90×10^{-2}	0.401567136	0.451	0.08262062	1	araphid1
Psammoneis	0.444644599	0.183	0.278596095	1	0.38077523	0.671	araphid1

obaidii							
Asterionella Formosa	0.479432935	8.05×10^{-2}	0.445734299	0.178	0.21128479	1	araphid2
Astrosyne radiata	0.743113847	2.41×10^{-6}	0.598559319	2.28×10^{-3}	0.64613157	3.55×10^{-4}	araphid2
Synedra acus	0.62298395	9.12×10^{-4}	0.439424521	0.205	0.49397144	5.57×10^{-2}	araphid2
***Licmophora* sp.**	0.682795027	6.72×10^{-5}	0.496816073	5.18×10^{-2}	0.4754451	8.89×10^{-2}	araphid2
Eunotia naegelii	0.493281213	5.68×10^{-2}	0.390963517	0.554	0.36885705	0.834	raphid
Cylindrotheca closterium	0.58102035	4.20×10^{-3}	0.453658639	0.149	0.53174079	1.98×10^{-2}	raphid
Seminavis robusta	0.574853202	5.18×10^{-3}	0.422466096	0.295	0.58854021	3.25×10^{-3}	raphid
***Entomoneis* sp.**	0.660163357	1.93×10^{-4}	0.586512136	3.49×10^{-3}	0.58024835	4.32×10^{-3}	raphid
***Fistulifera* sp. JPCC DA0580**	0.507540489	3.89×10^{-2}	0.436830462	0.217	0.43936038	0.205	raphid
Didymosphenia geminata	0.601524138	2.05×10^{-3}	0.505160094	4.15×10^{-2}	0.56247604	7.75×10^{-3}	raphid
Phaeodactylum tricornutum	0.599266452	2.22×10^{-3}	0.516731197	3.02×10^{-2}	0.5620021	7.87×10^{-3}	raphid
